# Psychometric properties of the Spanish version of the EuroQol-5D-5L in previously hospitalized COVID-19 survivors with long COVID

**DOI:** 10.1038/s41598-022-17033-1

**Published:** 2022-07-23

**Authors:** César Fernández-de-las-Peñas, Jorge Rodríguez-Jiménez, Paloma Moro-López-Menchero, Ignacio Cancela-Cilleruelo, Alberto Pardo-Hernández, Valentín Hernández-Barrera, Ángel Gil-de-Miguel

**Affiliations:** 1 Department of Physical Therapy, Occupational Therapy, Physical Medicine and Rehabilitation, Facultad de Ciencias de la Salud, Universidad Rey Juan Carlos (URJC), Avenida de Atenas s/n, Alcorcón, 28922 Madrid, Spain; 2 Consejería de Salud Pública, Comunidad de Madrid, Madrid, Spain; 3 Department of Public Health and Preventive Medicine, Universidad Rey Juan Carlos (URJC), Madrid, Spain

**Keywords:** Public health, Quality of life

## Abstract

The EuroQol 5-dimensions 5-levels (EQ-5D-5L) is a generic patient-reported outcome measures (PROM) used for evaluating health-related quality of life (HRQoL). No data on its psychometric properties in COVID-19 survivors is available. We aimed to describe internal consistency, test–retest reliability, and construct validity of the EQ-5D-5L in people with long-COVID. Ninety-three (n = 93) individuals previously hospitalized due to COVID-19 with post-COVID symptoms completed the EQ-5D-5L questionnaire twice one year after hospital discharge in a three-week interval. Internal consistency (Cronbach alpha and Omega value), test–retest reliability (kappa and ICC_2,1_) and construct validity (factor analysis), and floor/ceiling effects were calculated. No ceiling effect was observed in any dimension whereas the floor effect ranged from 53.76 to 94.62%. The overall Cronbach’s α value was 0.75 (95%CI 0.64–0.83) and the Omega ω value was 0.77 (95%CI 0.66–0.84), showing good internal consistency of the questionnaire. Further, Cronbach’s alpha values the of each dimension ranged from 0.63 to 0.77 whereas those for Omega values ranged from 0.70 to 0.79. The test–retest reliability of the total score was excellent (ICC_2,1_ 0.86, 95%CI 0.798–0.911). The agreement percentage ranged from 85.13 to 96.77%; but kappa coefficients ranged from fair (κ: 0.37) to good (κ: 0.61). The factor analysis showed factor loadings from 0.585 to 0.813 supporting good construct validity. The EQ-5D-5L has good psychometric properties to be used as a PROM to assess HRQoL in hospitalized COVID-19 survivors with long-COVID.

## Introduction

The severe acute respiratory syndrome coronavirus 2 (SARS-CoV-2) virus, responsible of causing coronavirus disease 2019 (COVID-19), mainly affects the respiratory system; however, multisystemic affection is present in most patients explaining the heterogeneity presentation of this condition. A multisystemic affectation would also explain the plethora of symptoms experienced after the acute phase, e.g., called long COVID or post-COVID^1^. Current evidence supports that almost 60% of COVID-19 survivors will experience post-COVID symptoms at least during the first months after infection^2^. The presence of long COVID leads to a decrease in health-related quality of life (HRQoL)^3^, a reduction in the daily living activities with loss of independency^4^, and an inability of returning to work^5^. A recent consensus definition of post-COVID includes function repercussion^6^: “…these symptoms generally have an impact on everyday function…” Accordingly, evaluation of function and HRQoL in people with long-COVID seems to be essential.

Patient-reported outcome measures (PROM) consist of generic or disease-specific self-reported questionnaires assessing different aspects of a condition. The Post-COVID-19 Functional Status (PCFS) Scale has raised as a disease-specific PROM evaluating the functionality of COVID-19 survivors^7^. In fact, the PCFS has been validated in the Spanish population^8^. Although the PCFS has good construct validity for classifying COVID-19 survivors according to their function, its association with HRQoL (EuroQol 5D-5L) has been found to be poor to moderate^9^.

Different generic or disease-specific PROM have been developed for evaluating the multidimensional concept of HRQoL^10^. The EuroQol 5-dimensions 5-levels (EQ-5D-5L) questionnaire^11^ is a generic widely instrument used to assess HRQoL in different populations^12^. In fact, the EQ-5D-5L has been used for evaluating HRQoL in the general population during the quarantine associated with the first COVID-19 outbreak^13^ and also in some studies including COVID-19 survivors with long-COVID^14^. Additionally, the fact that the EQ-5D-5L is the instrument used to generate utility-analyses by calculating the quality-adjusted life years (QALYs)^15^, its use in COVID-19 survivors will be needed for cost-utility studies in the future.

A recent systematic review found that the EQ-5D-5L exhibits excellent psychometric properties across different populations, conditions, and settings^16^. No previous study has investigated the psychometric properties of the EQ-5D-5L in COVID-19 survivors. This study aimed to describe internal consistency, test–retest reliability, and construct validity of the EQ-5D-5L questionnaire in a sample of COVID-19 survivors suffering from long COVID after hospitalization.

## Methods

### Participants

This study included patients who were hospitalized by acute SARS-CoV-2 infection during the first wave of the pandemic (March 20 to June 30, 2020) in an urban hospital of Madrid (Spain). All subjects attending to a specific post-COVID unit at the hospital between March 2021 and May 2021 were invited to participate in the study. Participants should have a SARS-CoV-2 infection diagnosis with real-time reverse transcription-polymerase chain reaction (PCR) assay of nasopharyngeal/oral swab samples and the presence of consistent clinical and radiological findings at hospital admission.

### COVID-19 collection data

Demographic data (e.g., age, gender, height, weight), clinical data (e.g., previous medical comorbidities), and hospitalization data (COVID-19 associated-onset symptoms experienced at hospital admission, intensive care unit [ICU] admission, days at hospital) were collected from hospital medical records.

Participants who agreed to participate were scheduled for a face-to-face interview by trained healthcare researchers. Participants were asked to report the presence/absence of symptoms after hospitalisation and whether the symptoms persisted at the time of the interview. It was emphasized that symptoms should have appeared after hospitalization. Participants were systematically asked for a predefined list of post-COVID symptoms such as dyspnoea, fatigue, anosmia, ageusia, hair loss, chest pain, palpitations, diarrhoea, skin rashes, brain fog, ocular/visual disorders, cough, and loss of concentration; however, they were free to report any persistent symptom that they considered relevant.

### Health-related quality of life

The EQ-5D-5L questionnaire includes five items assessing five health dimensions (e.g., mobility, self-care, daily life activities, discomfort/ pain, and depression/anxiety), each on with a five-level answer (1: no problems to 5: severe problems)^17^. Responses are converted into a single index number between 0 and 1 where 0 corresponds to a health state judged to be equivalent to death and 1 corresponds to optimal health, by applying crosswalk index values for Spain life^18^. In the current study, we used the validated version for the Spanish-speaking general population^19,20^.

All participants fulfilled the EQ-5D-5L during a first appointment performed a mean of 12 months (SD 5) after hospital discharge. Additionally, a standardized checklist including the following items was also collected: time needed for answering the EQ-5D-5L (as assessed with a digital chronometer), those questions not answered, and difficulty for understanding and answering the questionnaire. For test–retest reliability, participants fulfilled the EQ-5D-5L a second time after 3–4 weeks from the first appointment.

### Statistical analysis

Statistical analysis was performed with SPSS-software 23.0 (SPSS Inc, Chicago, IL, USA). Statistical significance was defined as a priori p-value < 0.05. Descriptive statistics (proportions, means, and standard deviations) were used to describe the study population. We tested the following properties of EQ-5D-5L questionnaire^21^ according to the COnsensus-based Standards for the selection of health Measurement INstruments (COSMIN)^22^:

1. *Internal consistency,* that is, the extent to which items measure the same underlying construct, was calculated by Cronbach alpha^23^ and the Raykow Omega Coefficient^24^. Values between 0.70 and 0.95 are considered to reflect good internal consistency.

2. *Reproducibility,* that is, the degree to which repeated test–retest measurements provide similar answers, concerns reliability, and agreement. We calculated the percentages of agreement and kappa coefficients to estimate test–retest concordance on each question of the EQ-5D-5L. We interpreted kappa values according to Landis and Kock^25^: excellent (0.81–1.0), good (0.61–0.80), moderate (0.41–0.60), fair (0.21–0.40), or poor (0.0–0.2). The test–retest reliability of the EQ-5D-5L total score was assessed with a two-way mixed-model, consistency-type intraclass correlation coefficient (ICC_2,1_). An ICC ≥ 0.70 was considered as good to excellent reproducibility^26^.

3. *Construct validity,* that is, the extent to which the score relate to other measures, was verified by means of a factor analysis by maximum likelihood setting a single factor. Both Kaiser–Meyer–Olkin measurement of sampling adequacy and Bartlett’s test of sphericity were used to assess if data were appropriate or not for factor analysis.

We also calculated the percentage of subjects achieving the highest (floor effect) and the lowest (ceiling effect) scores on each question of the EQ-5D-5L. In addition, chi-square tests were conducted to assess if there were significant differences by gender and by group age (grouped as < 45 years, 45–59 years, 60–69 years, and ≥ 70 years).

### Ethical approval

All methods were performed in accordance with the relevant guidelines and following the Helsinki Declaration. The current study was approved by the Local Ethic Committee of the Hospital Universitario Fundación Alcorcon (HUFA20/126).

### Informed consent

Participants were informed of the study objective and provided informed consent before their inclusion and before collecting any data.

## Results

### Participants

From an initial sample of 100 COVID-19 survivors attending to the post-COVID unit and who were invited to participate, seven refused to participate. A total of 93 (mean age: 57, SD: 14 years, 48.4% women) were finally included. The mean length of hospital stay due to COVID-19 was 7 days (SD 6.2). The most prevalent onset symptoms at hospital admission were fever (74.1%), myalgias (58.1%) and cough (35.5%). Each patient reported a mean of 2 (SD 1.6) COVID-19 onset symptoms at hospital admission. No patient required ICU admission. The mean number of pre-existing medical co-morbidities was 1.4 (SD 1). The mean number of post-COVID symptoms of each participant was 3.2 (SD 1.0). The features of the study population are summarized in Table 1.

**Table 1:** Demographic and clinical data of the sample (n = 93).

Age, mean (SD), years	57 (14)
Gender, male/female (%)	48 (51.6%)/45 (48.4%)
Weight, mean (SD), kg	80 (15)
Height, mean (SD), cm	166 (9.5)
**Symptoms at hospital admission, n (%)**	
Fever	69 (74.1%)
Myalgia	54 (58.1%)
Cough	33 (35.5%)
Dyspnoea	31 (33.3%)
Headache	30 (32.2%)
Diarrhoea	21 (22.6%)
Ageusia	21 (22.6%)
Anosmia	18 (19.3.5%)
Throat Pain	12 (12.9%)
Vomiting	7 (7.5%)
**Medical co-morbidities at hospital admission**	
Hypertension	32 (34.4%)
Diabetes	11 (11.8%)
Cardiovascular disease	6 (6.4%)
Asma	10 (10.7%)
Obesity	31 (33.3%)
Chronic obstructive pulmonary disease	4 (4.3%)
Rheumatological disease	4 (4.3%)
Other (cancer, kidney disease)	6 (6.4%)
Stay at the hospital, mean (SD), days	7.0 (6.2)
**Persistent post-COVID symptoms, n (%)**	
Pain symptoms	43 (46.25%)
Fatigue	30 (32.2%)
Hair loss	24 (25.8%)
Loss memory	24 (25.8%)
Anosmia	13 (14.0%)
Dyspnoea	12 (12.9%)
Brain fog	12 (12.9%)
Skin rashes	10 (10.7%)
Attention disorders	10 (10.7%)
Gastrointestinal disorders	9 (9.6%)
Tachycardia-palpitations	9 (9.6%)
Ageusia	9 (9.6%)
Ocular/vision disorders	8 (8.6%)
Throat pain	7 (7.5%)
Diarrhoea	5 (5.3%)
Cough	2 (2.1%)

### General data

The mean time for fulfilling the EQ-5D-5L was 47 (SD 25) seconds. All questions were properly answered all participants, and the questionnaire was perceived as easy and comprehensible for all patients. No ceiling effect was observed in any dimension, whereas the floor effect ranged from 53.76 to 94.62% (Table 2).

**Table 2:** Psychometric properties of the EQ-5D-5L questionnaire in COVID-19 survivors experiencing Long COVID.

Dimensions	Floor effect (%)	Ceiling effect (%)	Internal consistency (Cronbach α value)	Internal consistency (omega ω value)	Percentage agreement (%)	Kappa value	Factor loading
Mobility	76.34	0	0.66 (0.52–0.78)	0.77 (0.68–0.85)	92.83	0.55	0.813
Self-care	94.62	0	0.77 (0.67–0.85)	0.79 (0.70–0.86)	96.77	0.37	0.602
Daily life activities	80.65	0	0.67 (0.53–0.79)	0.78 (0.69–0.85)	94.62	0.61	0.751
Pain/Discomfort	53.76	0	0.63 (0.47–0.76)	0.70 (0.57–0.80)	85.13	0.51	0.722
Depression/anxiety	53.76	0	0.71 (0.59–0.81)	0.72 (0.60–0.81)	87.46	0.48	0.585

Data from Cronbach α value and omega ω value are mean (95% confidence interval).

**Figure 1:**
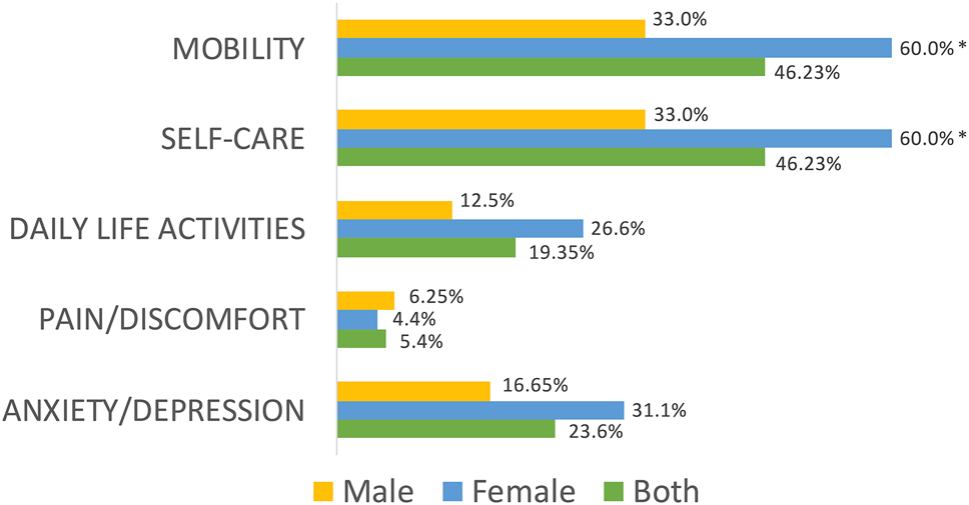
Distribution of the percentage of women and men exhibiting limitations on each domain of the EQ-5D-5L questionnaire. Asterisk: significant differences between men and women (P < 0.01).

**Figure 2:**
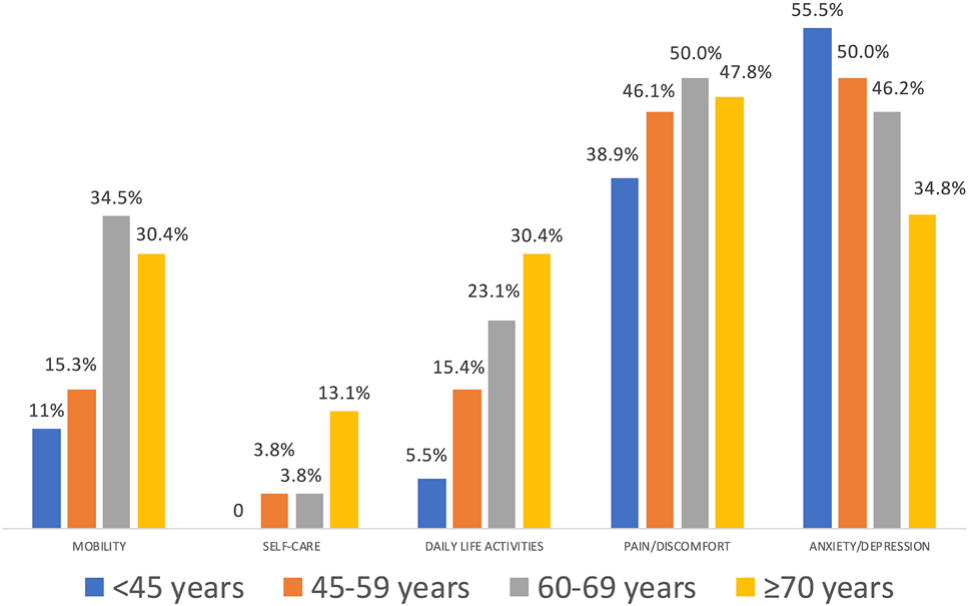
Distribution of the percentage of individuals exhibiting limitations on each domain of the EQ-5D-5L questionnaire by age group.

Those dimensions where a higher proportion of COVID-19 survivors experienced limitations were pain/discomfort (n = 43, 46.25%) and anxiety/depression (n = 43, 46.25%), followed by mobility (n = 22, 23.6%), daily life activities (n = 18, 19.35%) and self-care (n = 5, 5.4%). A greater proportion of women exhibited limitations on pain/discomfort and anxiety/depression dimensions when compared with men (P = 0.01). No sex differences in mobility (P = 0.101), self-care (P = 0.700), and daily life activities (P = 0.084) dimensions were observed (Fig. 1). No significant differences across the aged-groups were found in any dimension (Fig. 2): mobility (P = 0.182), self-care (P = 0.270). daily life activities (P = 0.212), pain/discomfort (P = 0.905), and anxiety/depression (P = 0.574).

### Internal consistency

The overall Cronbach’s α value of the EQ-5D-5L questionnaire was 0.75 (95%CI 0.64–0.83) whereas the overall Omega ω value of the EQ-5D-5L questionnaire was 0.77 (95%CI 0.66–0.84), showing good internal consistency of the questionnaire. However, Cronbach’s alpha values of each dimension ranged from 0.636 (pain/discomfort) to 0.769 (self-care). The only two single dimension showing good internal consistency (Cronbach’s α > 0.70) were self-care and anxiety/depression (Table 2). On the contrary, Omega ω values of each dimension was all ≥ 0.70.

### Test/retest reliability

The EQ-5D-5L questionnaire was administered twice with a mean difference of 25 (SD 2) days. The mean score was 0.86 (SD 0.16) at the first appointment and 0.90 (SD 0.12) at the second appointment. The test–retest reliability of the total score was excellent (ICC_2,1_ 0.86, 95%CI 0.798–0.911).

The agreement percentage was high, ranging from 85.13% (pain/discomfort item) to 96.77% (self-care dimension); nevertheless, kappa coefficient ranged from fair (κ: 0.37 self-care dimension) to good (κ: 0.61, daily life activities dimension) (Table 2).

### Construct validity

The results of the factor analysis showed that the Kaiser–Meyer–Olkin value was 0.73, indicating that the sample size was appropriate for the principal component analysis. The Bartlett’s test of sphericity (P = 0.00) indicated that the variables were correlated and, hence, suitable for factor analysis. The factor analysis showed factor loadings ranging from 0.585 (anxiety/depression) to 0.813 (mobility) for all dimensions (Table 2).

## Discussion

The presence of post-COVID symptoms (i.e., long COVID) provokes a decrease in HRQoL^3^. The heterogeneity of long COVID involves a multisystemic affectation which needs a complete evaluation. Generic and specific PROM are commonly used for evaluating HRQoL. To date, no disease-specific PROM evaluating HRQoL is available for long COVID^3^. We aimed to determine the psychometric properties of the Spanish version of the EQ-5D-5L in previously hospitalized people with long COVID. The results showed that the Spanish version of the EQ-5D-5L adequately fulfill the properties of floor and ceiling effects—as well as those of validity and reliability- with high Cronbach alpha values. In fact, a recent systematic review reported that the EQ-5D-5L exhibits excellent psychometric properties across a broad range of populations, conditions and settings, but individual dimensions exhibited test-rest instability^16^. Similar results were observed in our sample of COVID-19 survivors with long COVID since test–retest reliability of each dimension (*k*) ranged from fair to good, in opposite with an excellent reliability (ICC_2,1_) of the EQ-5D-5L total score.

Our sample of individuals with long COVID experience a mean of 3 post-COVID symptoms one year after hospitalization, supporting that these symptoms are long-lasting. The most common post-COVID symptoms were pain and fatigue. Accordingly, the most affected dimensions in our sample of individuals with long COVID were pain/discomfort and anxiety/depression followed by mobility in agreement with previous studies^27,28^. In fact, women exhibited more limitations in these dimensions than men, supporting current assumptions that female sex is more affected by long COVID^29^. Our EQ-5D-5L total scores were similar to those previously seen in a Spanish population hospitalized in the previous 12 months^19^. The review conducted by Poudel et al. reported EQ-5D-5L mean total scores ranging from 0.612 to 0.714 in those studies evaluating HRQoL in people with long COVID^14^. Discrepancies between previous studies and current data may be explained by the heterogeneous follow-up periods those studies included in Poudel et al. review were conducted in the first 4 to 12 weeks from the onset of symptoms^14^ whereas our sample was assessed one year after hospitalization.

Other PROMs assessing HRQoL, e.g., SF-12^30^, 15D^31^, or SF-36^32^, have been also used in individuals with post-COVID symptoms. All these studies reported the same results, people with long-COVID exhibit reduced HRQoL. The EQ-5D-5L is a simple and easily comprehensive PROM for patients which takes just less than 60 s to be fulfilled. Considering that several COVID-19 survivors may suffer from post-COVID cognitive problems, e.g., brain fog or concentration loss, easy and quick PROMs should be more recommended.

Although the EQ-5D-5L includes five dimensions (e.g., mobility, self-care, usual activities, pain/discomfort, and anxiety/depression), the large and heterogeneity variation in HRQoL that COVID-19 survivors suffering from long COVID experience, a disease-specific PROM for this condition is needed. In fact, the only disease-specific PROM for COVID-19 is the PCFS, which has shown good validity and reliability^7^, although its correlation with HRQoL (as assessed with the EQ-5D-5L) was poor^9^. The evaluation of long COVID should be comprehensive by including disease-specific (i.e. PCFS) but also generic (e.g., EQ-5D-5L) PROMs.

The main limitation of the present study is the inclusion of just hospitalized COVID-19 survivors and the relatively sample size. It is possible that the psychometric data of the EQ-5D-5L would be slightly different in non-hospitalized subjects. Further, no individual required ICU at hospital, which also limit extrapolation of the results. It is possible that those limitations identified by the EQ-5D-5L could have shifted towards more functional impairment in individuals requiring ICU admission. In addition, our sample of COVID-19 survivors was assessed one year after hospital discharge, a long-term follow-up assessment. We did not have data about HRQoL before the infection or during the first months after hospitalization. In fact, our scores of the EQ-5D-5L were higher (reflecting better HRQoL) than those previously reported by other studies evaluating HRQoL in individuals with long COVID at shorter follow-up periods (from 4 to 12 weeks after infection), probably because HRQoL tends to improve with time. Future studies should investigate longitudinal evolution of EQ-5D-5L to identify the potential responsiveness to change of this questionnaire. In fact, Hedge et al. has recently observed an improvement in HRQoL (as assessed with the EQ-5D-5L) in a sample of COVID-19 survivors after treatment^33^. Finally, we did not either include any objective measure of physical functioning that could be related to HRQoL.

## Conclusion

This study results suggest that the EQ-5D-5L questionnaire has good psychometric properties to be used as a generic PROM to measure HRQoL in previously hospitalized COVID-19 survivors with long COVID. This assumption is based on a limited sample of individuals with long COVID. Accordingly, further studies including larger sample sizes are now needed to further determine current results.

## Data Availability

All data derived from this study are presented in the text.
